# Impact of COVID-19 Disease Control Committee (CDCC) policies on prevention of the disease using Bayes network inference in west of Iran

**DOI:** 10.1186/s12889-023-16879-y

**Published:** 2023-10-16

**Authors:** Ali Reza Soltanian, Roya Ahmaddoost-razdari, Hossein Mahjub, Jalal Poorolajal

**Affiliations:** 1 School of Public Health, Modeling of Noncommunicable Diseases Research Center, Hamadan University of Medical Sciences, Street of Shahid Fahmideh, Hamadan, 6517838687 Iran; 2 Department of Biostatistics, School of Public Health, Hamadan University of Medical Sciences, Hamadan, 6517838687 Iran; 3 Research Center for Health Sciences, Hamadan University of Medical Sciences, Hamadan, 6517838687 Iran

**Keywords:** SARS-CoV-2, Bayes theorem, Health policy, Surveillance, Emergency management

## Abstract

**Background:**

The start of the COVID-19 pandemic was an emergency situation that led each country to adopt specific regional strategies to control it. Given the spread of COVID-19 disease, it is crucial to evaluate which policy is more effective in reducing disease transmission. The purpose of this study was to determine the impact of policies made by COVID-19 Disease Control Committee (CDCC) to reduce the risk of the disease in Hamadan province.

**Methods:**

In the observational study, the data were extracted from three sources in Hamadan, west of Iran; first, the session reports of CDCC; second, information on periodic evaluations conducted by the primary health care directory in Hamadan from April to August 2021 and third, expert panel opinion. Bayes network analysis was used to determine the effect of each policy on mortality rate by GeNIe software version 2.2.

**Results:**

Among the policies adopted by CDCC in Hamadan, seven policies, i.e., vaccination, limiting gatherings, social distancing, wearing a mask, job closure, travel restriction, and personal hygiene had the most impact to prevent the spread of COVID-19, respectively. In this study, the prevalence of the disease was 17.64% with the implementation of these policies. Now, if all these policies are observed 30% more, the prevalence will decrease to 14.18%.

**Conclusion:**

This study showed that if the seven policies (i.e., vaccination, limiting gatherings, social distancing, wearing a mask, job closure, travel restriction, and personal hygiene) are followed simultaneously in the community, the risk of contracting the disease will be greatly reduced. Therefore, in the pandemic of infectious diseases, such policies can help prevent the spread of the disease.

## Introduction

COVID-19 is one of the most contagious infectious diseases in the world. The SARS-CoV-2 was first detected in December 2019 in Wuhan, Hubei province, China [1]. The virus rapidly disseminated to various regions the globe as China sought to reduce the spread of the disease through non-drug measures [2]. Up to May-3–2022, according to the website of the Corona Hopkins Virus Resource Center,^1^ the global tally revealed 443,777,811 cases of infection and 5,989,860 reported fatalities [3]. Mortality from the disease depends on factors such as population size, prevalence, age, sex, and immune system [2, 3].

According to the previous studies, Hamadan province was recognized as high-risk areas among its neighboring provinces (i.e., Lorestan, Kurdistan, Kermanshah, Markazi, Zanjan and Qazvin) [4, 5].

Although the conventional diagnostic test for COVID-19 is Real-time PCR, it is not completely accurate in diagnosing COVID-19 disease [6]. For this reason, people with the following conditions need to receive the necessary medical care: i) have clinical symptoms similar to those of COVID-19, ii) travel in contaminated areas, iii) have close contact with suspects [7]. Governments have adopted different policies and strategies to control and reduce the risk of COVID-19 infection. For example, Japan [8] abolished the entry of foreigners from 73 countries and imposed an emergency state throughout the country, so that people were required to observe social distancing, wash their hands, wearing a mask and minimize indoor activities. As a result of ongoing the pandemic, all concerts had to be canceled and schools were forced to shut them.

In Britain, the government initially refrained from imposing pandemic restrictions, hence the risk of infection, as well as mortality, increased. Then, decided to implement strict policies [9].

To deal with the COVID-19 pandemic crisis, South Korea quickly developed diagnostic kits and performed the necessary tests [10]. The strategies helped the government identify infected people and quarantine them quickly. Furthermore, the South Korean government has introduced complimentary smartphone applications designed to notify individuals through emergency text messages [10]. In terms of the policy to reduce the prevalence of COVID-19, China as the first country where the disease emerged, taking measures such as quarantining people with the disease or even suspected, observing anti-viral coverage (e.g., masks, gloves, and goggles), building new hospitals and quarantining contaminated areas [11].

Vietnam has also been one of the countries at risk of COVID-19. The first measures of the Vietnamese government to prevent the spread of the disease were the cancellation of all flights from Vietnam-infected areas and vice versa, the use of masks in public places, widespread information through the national media [12].

Considering the actions of populous and high income countries such as China, Japan, South Korea, as well as Vietnam, it can be noted that planning, rapid response, clinical care, public awareness, and public cooperation with the government to reduce the prevalence of pandemics are very important. On the other hand, it should be emphasized that personal hygiene measures such as washing hands with appropriate detergents, wearing a face mask, social distancing, using disinfectants (e.g., alcohol), etc. can control the rate of spread of infectious-respiratory diseases such as coronavirus [13]. The policies mentioned in the first step reduce the spread of the disease, but due to the presence of asymptomatic patients, these methods may not be completely effective [14]. Moreover, delays in communicating and implementing policies, lifting restrictions early, or prolonging the pandemic period too long can lead to many negative economic and social consequences [13].

Given the spread of COVID-19 disease, it is crucial to evaluate which policy is more effective in controlling and reducing disease transmission. Various models have been developed to determine the risk of COVID-19 disease, but no model can evaluate the general policies of a regional government to prevent the occurrence of this disease. Therefore, the purpose of this study is to determine the impact of the policies and plans made by “*the COVID-19 Disease Control Committee (CDCC)*” in Hamadan province to control and reduce the risk of coronavirus.

## Methods

This observational study was conducted throughout the period between April 2 to Aguste 21, 2021. This period was five months before the fifth wave of the COVID-19 pandemic in Hamadan, west of Iran.

### Data collection methods

The data used in the study were recruited from three sources: first, the reports of CDCC’s sessions; second, information on periodic surveys conducted by the Primary Health Care (PHC) directory in Hamadan province; and third, expert panel opinion. This study was performed in accordance with the declaration of Helsinki, and informed consent was obtained from all subjects and/or their legal guardian. The study was approved by Research Ethics Committee of Hamadan University of Medical Sciences (IR.UMSHA.REC.1400.329). In Iran, the National Committee for Dealing with the COVID-19 pandemic was formed in March 2018 with the aim of policymaking to prevent and control the disease. After that, this committee was formed throughout the provinces of Iran. These committees took the crucial decisions and restrictions on a provincial basis. For this reason, firstly, we reviewed 25 reports of this committee in a five-month period before the fifth wave of COVID-19. We readout all the decisions in a checklist.

During this process, 91 restrictions were noted in the Hamadan CDCC's reports. After unifying the duplicate restrictions and removing seven restrictions that were only proposed once throughout the period between April 2 to Aguste 21, 2021, finally, seven restrictions were considered as the fundamental policies to prevent and control the COVID-19 pandemic. Notice, omitted restrictions occurred only once during this period, therefore, their prior probability could not be obtained for running the Bayes network model.

**Fig. 1:**
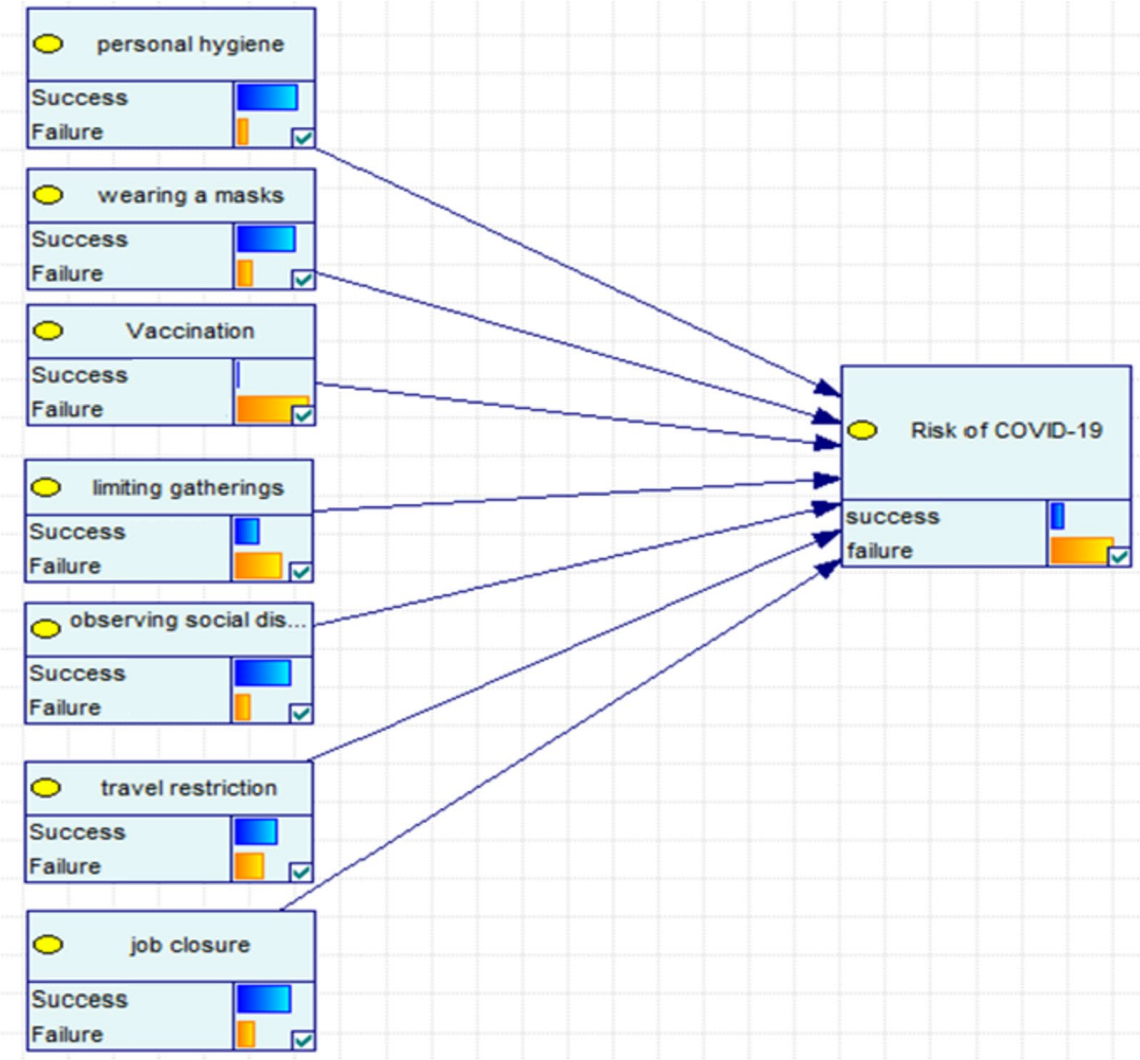
A conceptual model based on the seven policies (or decisions) of the COVID-19 Disease Control Committee in Hamadan province

Without losing the generality of the analysis, the seven restrictions were classified into three independent components according to the expert panel suggestion (see Fig. 1 and Table 1). The first component included two policies (i.e., social distancing, limiting gatherings); the second component included three policies (i.e., personal hygiene, wearing a mask, vaccination status) and the third component included two policies (i.e., travel restrictions, school closures).

**Table 1:** Extraction and unifying of CDCC’s policies in Hamadan province during COVID-19 pandemic

Row	Decisions	Model variables	Model No
1	social distancing	Model 1 = social distancing and limiting gatherings	(1)
2	limiting gatherings		
3	personal hygiene	Model 2 = personal hygiene, wearing mask and vaccination	(2)
4	wearing a mask		
5	Vaccination		
6	travel restriction	Model 3 = travel restriction and job closure	(3)
7	job closure		
8	social distancing	Model 1&2 = social distancing, limiting gatherings, personal hygiene, wearing a mask, and vaccination	(4) = Combination of Models (1) and (2)
9	limiting gatherings		
10	personal hygiene		
11	wearing a mask		
12	Vaccination		
13	social distancing	Model 1&3 = social distancing, limiting gatherings, travel restrictions, and job closure	(5) = Combination of Models (1) and (3)
14	limiting gatherings		
15	travel restriction		
16	job closure		
17	personal hygiene	Model 2&3 = personal hygiene, wearing a mask, vaccination, travel restriction, and job closure	(6) = Combination of Models (2) and (3)
18	wearing a mask		
19	Vaccination		
20	travel restriction		
21	job closure		
22	social distancing	Model 1&2&3 = social distancing, limiting gatherings, personal hygiene, earing a mask, vaccination, travel restriction, and job closure	(7) = Combination of Models (1), (2) and (3); i,e., the conceptual model
23	limiting gatherings		
24	personal hygiene		
25	wearing a mask		
26	vaccination		
27	travel restriction		
28	job closure		

The second part of the data was prepared from the PHC directory in Hamadan, which included variables such as the proportion of personal hygiene, the proportion of wearing a mask, social distancing rate, and the vaccination rate. This information has been determined by periodic surveys in customer service centers, industries, public places, and food preparation and distribution centers, etc. This information was collected monthly by the PHC directory. During the COVID-19 pandemic, the chairs were marked in the stands and arranged with a distance of 1.5 m. The PHC staffs randomly visited some of these places and based on the people were sitting on the chairs, the physical distance and wearing a mask rates were estimated. People were also asked to estimate the level of compliance with personal hygiene. In this study, the vaccination proportion was the ratio of people who received the second dose of the vaccine to the eligible population.

In the third part, the knowledge and experience of an expert panel (*n* = 10) has been used to complete the conditional probability table using the Delphi method as follow. Panel members opinions were used to select same policies and categorize them in Delphi. In the first, all policies (i.e., 91 policies/restrictions were noted in the Hamadan CDCC's reports) were provided to them in the form of a list. They commented on the importance and ability to measure those restrictions. After collecting their opinions, we unified and categorized the policies to 28 policies in seven categories. Second, we showed them the new results and polled them again. Finally, their approval was obtained in all cases. This expert panel works in various fields contains the president of Hamadan University of Medical Sciences as the secretary of the COVID-19 disease control committee, the vice-chancellor for public health, the vice-chancellor for research and technology, the vice-chancellor for clinical administration, a number of infectious disease physicians, epidemiologists, and the director of infectious diseases. According to the Delphi method, the extracted variables were provided to the expert panel to review and apply their opinions.

The opinions of the expert panel were used in two steps to unify and remove duplicate policies. The panel members examined 91 decisions and some of them were excluded from the study due to the lack of clarity for the following reasons: i) adopting some of them only once during the study, ii) some decisions cannot be measured, iii) the same process of the policies during the study as an ineffective variable. For example, "restriction of inter-provincial travel" and "restriction of night traffic" according to the opinion of the panel members, were unified and entered into the study in the form " travel restriction". Then, in order to estimate the conditional probability table, checklists were provided for the panel members and finally, the average of their opinions was used.

### Statistical analysis

Bayes network model (e.g., given data *x*, parameter $\theta$, prior probability $p\left(\theta \right)$ and likelihood $p\left(x|\theta \right)$ to compute a posterior probability $p\left(\theta |x\right)\propto p\left(x|\theta \right)p\left(\theta \right)$) has been used to determine the effect of variables or components on the risk of COVID-19 by GeNIe software version 2.2.

In the first stage of this network, we obtained the input information as mentioned in “ Data collection methods” Section. In the second step, we obtained conditional probability table (i.e., $p(x|\theta ))$ values with the guidance of panel members and given data (i.e., input information).

Bayes network diagrams were drawn under binomial-prior probabilities and then the network posterior probabilities were updated. The conceptual model in this study is shown in Fig. 1 based on the available data (CDCC's policies) and expert panel opinion.

In order to evaluate the accuracy of each of the fitted models, the root mean square error (RMSE) statistical index has been used as follows.

$$\mathrm{RMSE}=\sqrt{\frac{1}{\mathrm{N}}\sum_{\mathbf{i}=1}^{\mathbf{N}}{({\mathbf{y}}_{\mathbf{i}}-{\mathbf{y}}_{\mathbf{i}}^{\mathrm{^{\prime}}})}^{2}}$$

where ${\mathbf{y}}_{\mathbf{i}}^{\mathrm{^{\prime}}}$ are the estimated values, ${\mathbf{y}}_{\mathbf{i}}$ are the observed values, N is the number of observations [15]

Then, using Fussell-Vesely Importance Measure (FV), the importance of each of the variables (policies) was calculated separately in the Bayes network model.

The variable importance criterion is actually the total risk of the fitted model minus the risk value of the desired variable divided by the total risk of the fitted model [16].

$$\mathrm{FV}=\frac{\mathrm{P}\left(\mathrm{Total}\right)-\mathrm{P}({\mathrm{x}}_{\mathrm{i}}=0)}{\mathrm{P}\left(\mathrm{Total}\right)}$$

## Results

The crucial data for this study were collected from April to August 1400 and 25 CDCC’s reports were verified and readout. Throughout the CDCC’s reports, 91 variables (i.e., policies) were extracted. According to the expert panel opinion, after removing duplicate decisions and unifying similar decisions, seven decisions were finally approved from those variables (Table 1).

To perform the Bayes network model and estimate the risk of COVID-19 under the policies adopted by the CDCC, the prior and posterior probabilities of each variable were obtained. In this study, first, the risk of COVID-19 disease was obtained separately for each model (see models 1–3, Table 2). Second, the model was entered into the Bayes network model in pairs and the risks were measured again (see models 4- 6, Table 2). Finally, the conceptual model (see Fig. 1 and model 7) was performed based on the inclusion of three main models in the Bayes network model, and then the risk of COVID-19 disease was obtained. A risk assessment by combining the three models as well as measuring the impact of each of the main models on the risk of COVID-19 is shown in Table 2.

**Table 2:** Effect of CDCC’s policies on risk of COVID-19 using Bayes network model

Model	Policies and components in the Bayes network model	Prior Probability	Risk of infection (%)	Risk of infection (%)^a^	Risk of infection (%)^b^	Risk of infection (%)^c^
First	Variable 1: observing social distancing	0.779	42.06	38.85	35.63	32.81
	Variable 2: limiting gatherings	0.35				
Second	Variable 1: personal hygiene	0.834	31.65	27.19	23.00	22.11
	Variable 2: wearing a mask	0.783				
	Variable3: vaccination	0.346				
Third	Variable 1: travel restriction	0.6	31.85	30.47	29.24	28.12
	Variable 2: job closure	0.75				
Fourth	Variable 1: observing social distancing	0.779	18.87	17.52	16.17	15.22
	Variable 2: limiting gatherings	0.35				
	Variable 3: personal hygiene	0.834				
	Variable 4: wearing a mask	0.783				
	Variable 5: vaccination	0.346				
Fifth	Variable 1: observing social distancing	0.779	20.98	19.46	17.77	16.00
	Variable 2: limiting gatherings	0.35				
	Variable 3: travel restriction	0.6				
	Variable 4: job closure	0.75				
Sixth	Variable 1: personal hygiene	0.834	16.25	15.19	14.12	13.37
	Variable 2: wearing a mask	0.783				
	Variable 3: vaccination	0.346				
	Variable 4: travel restriction	0.6				
	Variable 5: job closure	0.75				
Seventh	Variable 1: observing social distancing	0.779	17.64	16.38	15.13	14.18
	Variable 2: limiting gatherings	0.35				
	Variable 3: personal hygiene	0.834				
	Variable 4: wearing a mask	0.783				
	Variable 5: vaccination	0.346				
	Variable 6: travel restriction	0.6				
	Variable 7: job closure	0.75				

^a^The risk reduction of COVID-19 in the community, when the CDCC’s policies are implemented 10% more

^b^The risk reduction of COVID-19 in the community when the CDCC’s policies are implemented 20% more

^c^The risk reduction of COVID-19 in the community when the CDCC’s policies are implemented 30% more

After fitting the first model (i.e., observing the social distancing and the limiting gatherings imposed by the CDCC), the risk of COVID-19 disease is estimated at 42.06% (Table 2). In a situation where the two mentioned measures increase by 10% at the same time, the risk of COVID-19 disease decreases from 42.06% to 38.85%. In other words, if social distancing and limiting gatherings increase by 10%, the risk of contracting this disease will decrease by 3.21%. As can be seen in model (1) in Table 2, with an increase of 20% and 30% of the prior probabilities in the variables, the spread risk of COVID-19 disease is estimated at 35.63% and 32.81%, respectively**.**

According to the Bayes network results in the second model, with the observance of the personal hygiene, wearing a mask and vaccination the risk of COVID-19 disease is 31.65%. In this model, if the three factors are followed by 10% more by people, the risk of contracting the disease will decrease by 4.46%. In other words, this rate will decrease from 31.655% to 27.195.

Next, model 3 was implemented independently by introducing two policies called traffic restrictions and job closures into the Bayes network model. The model shows that these two policies alone can reduce the risk of SARS-CoV-2 to 31.85%.

As the results of Table 2 show, with a 10% increase in these implemented policies, the risk of contracting the COVID-19 will decrease to 1.36% in this region.

Model 4, which includes social distancing, limiting gatherings, personal hygiene, wearing a mask and vaccination, shows that by applying the five policies the risk of COVID-19 is about 18.87%. This study shows that if the central government can increase the intensity of these five policies by 30%, the risk of COVID-19 will decrease from 18.87% to 15.22% (Table 2).

The results of this study show that by implementing the four policies, i.e., social distancing, limiting gatherings, travel restriction and job closure, the risk of the new corona-virus infection will be 20.98% in the area (Model 5, Table 2). In the study, if the application of these restrictions can be increased to 30%, the risk of contracting COVID-19 will be 16%, which is approximately a 5% reduction.

In the sixth stage of Bayes network modeling (Model 6), by entering restrictions such personal hygiene, wearing a mask, vaccination, travel restriction and job closure, the risk of the disease was 16.25%. The results of this model show that with a 30% increase in the application of such restrictions, the risk of the disease can only be reduced by 2.88% (Model 6 and Table 2).

Model 7 is a conceptual model that includes components 1, 2 and 3 in Table 2. In this model, by applying all the conditions (seven policies) based on the CDCC decisions, the risk of COVID-19 was estimated at 17.64%. Now, in this model, by applying all the conditions according to the decision of the COVID-19 Disease Control Committee more comprehensively (30% increase), the risk of contracting the disease is reduced to 3.46%.

In this study, RMSE was used to select the best Bayes network model as a goodness of fit index. According to the results, model 7, which is the final model of the study and consists of all the CDCC's policies, has the lowest RMSE = 0.193, so it is known as the best fitted model (Table 3). In the next rank, the sixth model, which includes personal hygiene, vaccination, wearing a mask, travel restriction, and job closure, was recognized as the second most efficient model (RMSE = 0.492).

**Table 3:** Description of Bayes models, good-ness of fit and importance rate

Rank	Model	Variable in model	FV	RMSE
1	Seventh	Variable 1: vaccination	0.264	0.193
		Variable 2: limiting gatherings	0.089	
		Variable 3: observing social distancing	0.059	
		Variable 4: wearing a mask	0.055	
		Variable 5: job closure	0.037	
		Variable 6: travel restriction	0.036	
		Variable 7: personal hygiene	0.015	
2	Sixth	Variable 1: vaccination	0.431	0.492
		Variable 2: wearing a mask	0.204	
		Variable 3: job closure	0.054	
		Variable 4: travel restriction	0.052	
		Variable 5: personal hygiene	0.021	
3	Fourth	Variable 1: vaccination	0.309	
		Variable 2: limiting gatherings	0.136	
		Variable 3: observing social distancing	0.075	
		Variable 4: wearing a mask	0.07	
		Variable 5: personal hygiene	0.022	
4	Fifth	Variable 1: limiting gatherings	0.148	0.760
		Variable 2: travel restriction	0.119	
		Variable 3: job closure	0.068	
		Variable 4: observing social distancing	0.062	
5	Third	Variable 1: travel restriction	0.147	0.980
		Variable 2: job closure	0.075	
6	First	Variable 1: limiting gatherings	0.290	2.389
		Variable 2: observing social distancing	0.171	
7	Second	Variable 1: vaccination	0.205	2.820
		Variable 2: personal hygiene	0.201	
		Variable 3: wearing a mask	0.118	

**Fig. 2:**
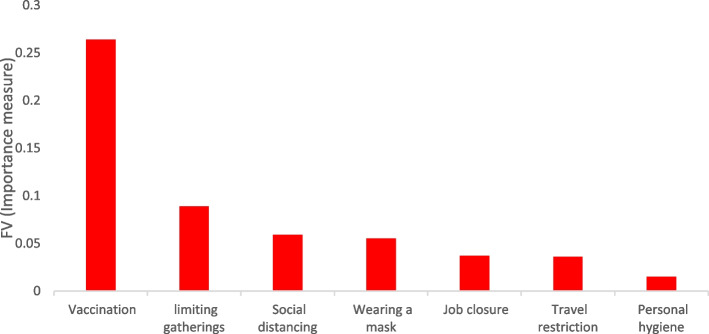
The importance rate of the preventive policies of the COVID-19 based on Fussell-Vesely index

In this study, the FV-index was used to determine the importance of each policy on the control of the spread risk of COVID-19, whose values are shown for each model in Table 3. According to this index in the seventh model, vaccination has been found as the most important variable, and it was followed by limiting gatherings, observing social distancing, wearing a mask, job closure and personal hygiene, respectively (Table 3 and Fig. 2).

## Discussion

The main purpose of this study was to evaluate the policies adopted by the COVID-19 Disease Control Committee (CDCC) in Hamadan province to control and reduce the risk of SARS-CoV‐2. Likewise, the present study quantitatively assessed the impact of regional government policies and decisions on the risk of COVID-19 disease by using the Bayes network model. Our literature showed that many studies have been conducted regarding the relationship between drug intervention and risk factors with the COVID-19. No study demonstrating the impact of government policies on reducing prevalence rates through statistical methods has been discovered. Consequently, this study distinguishes itself as a unique and unparalleled contribution. In this study, furthermore to determining the amount and priority of the effect of each policy on the risk of the disease, we committed a set of policies that can have the greatest control effect. Another strength of this study is that control policies were mentioned based on priority, while no such prioritization was introduced in any report. The implementation framework of this study can be used as a comprehensive model in other pandemics.

First, we extracted seven fundamental policies based on CDCC’s reports in Hamadan province and used the expert panel opinion for a qualitative evaluation. Then, we divided the seven situations into three models according to their conceptual and functional similarity and performed modeling. It is obvious that the more restrictions are applied to control the disease of COVID-19 during the pandemic, the lower the risk of contracting the disease.

Unlike China [11] and Japan [8], which were able to prevent the rapid spread of COVID-19 by applying two or three restrictions such as lockdown and personal hygiene, but this study showed that if several effective measures are applied simultaneously, we will reach a more favorable result in this way. In other words, although each of the policies announced in this study alone can control the amount of the infection rate, but the identification and evaluation of a combination of adopted policies and strategies are effective in reducing the infection rate.

In this research, seven Bayes network models were implemented with different combinations of the CDCC's policies. After careful evaluation, the seventh model has been chosen as the most optimal model due to its superior goodness of fit index, i.e., RMES, among them. In second place, with a slight difference, the sixth model, which includes the strategies of personal hygiene, wearing a mask, vaccination, travel restriction and job closure, has been shown to be a second predictive model.

To investigate the effect of the implementation of the seven policies included in the seventh model on predicting the probability of contracting COVID-19, the amount of each policy was increased to 30%, and then the risk of the infection was increased.

The result of the seventh model clearly states that the CDCC's policies are able to reduce the risk of infection to 3.72%, when restrictions are increased by 30%. Therefore, comply with personal hygiene, wearing a mask, vaccination, travel restriction, job closure, observing social distancing and limiting gatherings can greatly reduce the risk of contracting COVID-19.

In the conducted study, vaccination emerged as a paramount element in effectively curtailing the transmission of the COVID-19 disease. Although our study was conducted before the fifth peak in Hamadan province, and also the alpha and beta variant had been released among the population, but expanding public vaccination was more effective than other CDCC’s preventive policies. In England, researchers found that with two vaccinations either BNT162b2 or ChAdOx1nCoV-19, the chance of contracting delta-COVID-19 can be reduced [17]. In the USA also showed that when COVID-19 is spread at a high level in an area, vaccination can increasingly and effectively reduce the spread and deaths caused by it [18]. Our findings the same as the other studies, showed that vaccination is more effective than any other restrictive policy in reducing the spread of the disease. Hence, it is strongly recommended that in emergency pandemics, individuals ought to prioritize vaccination activities if a viable vaccine becomes accessible.

Our literature reviews show that social distancing is an effective way to contain the spread of an infectious disease, especially when little is known about the virus and no vaccine or other drug intervention is available [19]. Social distancing and isolation along with other non-pharmacological measures such as hygiene and wearing a mask have a direct impact on infection rates and thus on the spread of the virus [20, 21]. In the present study, a notable observation has been made regarding the efficacy of social distancing as the third most crucial measure in disease control.

Social distancing and wearing a mask were not identified as the first and second prevention factors in this study, because the government was not able to completely lockdown, and on the other hand, people were not able to fully provide masks due to financial problems. A study conducted in January 2021 in Hamadan showed that 13% of people were not wearing a mask in public passengers, and social distancing was less than the standard measure in Hamadan [22].

Of course, it should be emphasized that the strict implementation of social distancing may cause serious economic and psychological damage, which has been mentioned in some studies [19].

Adopting such lite preventive policies is more suitable for lower/middle income countries and low-income areas than the method of mandatory home quarantine, which happened in Hong Kong and other high-income countries [23]. It is emphasized that home quarantine is very expensive and it is impossible for governments in long time.

In addition to the previously mentioned restrictions, job closures can also play a role in reducing the spread of the virus. In Hamadan province, job closures were implemented according to a division schedule and changed according to the increase or decrease in the prevalence of SARS-CoV-2. This action was an appropriate strategy in that period. In Italy, we also found that business closures were done periodically, and they were able to reduce the spread of the disease and mortality. In Italy, as in our study, it was noted that although closing businesses is effective in controlling the spread of the respiratory disease COVID-19, it should be done selectively and periodically [24].

One of the effective restrictions on reducing the spread of the virus is limiting travel. In China [11], where the implementation of travel restrictions was effective in reducing COVID-19 unlike Hamadan province, which may be due to the following reasons: First, in China, the restrictions were implemented as a complete lockdown, while in this province, people's movement was restricted only hours within a day, and business closures were also applied selectively by the regional government. To clarify, in this study, commuting and some jobs were maintained due to economic and support problems during the COVID-19 pandemic, and people went there to provide their daily life needs and receive some services. Secondly, family parties were not held in China and people were completely aligned with the government in this regard. But this was not possible due to family parties and people's non-cooperation with the regional government. The application of the two policies of travel restrictions and job closure indirectly have the effect of the two policies of observing social distancing and limiting gatherings continuously. In the present study, although social distancing and wearing masks was relatively acceptable in indoor places, it seems to be much less in public crossings and streets.

Comparing the policies adopted in this study with countries such as China [11], South Korea [10], Japan [8], and Vietnam [12], which have been able to prevent SARS-CoV-2 on a large scale, shows that compliance with personal hygiene, early detection of carriers and provision of free sanitary supplies can prevent the disease quickly. In this study, the level of personal hygiene was considered to be about 83%, but based on the Bayes network model, it was identified as the last effective policy in preventing COVID-19. Of course, this result is not far from expected. Notice, during an infectious pandemic, the most important policy is to break the chain of disease transmission such as vaccination, quarantine and business closure. In this research, we found that measures such as vaccination, creating individual restrictions (e.g., staying away from large gatherings, closing jobs and avoid unnecessary travel), observing health principles (e.g., physical distance and wearing a mask) are very important in reducing the COVID-19 prevalence rate.

### Limitations

We had several limitations in this study. First, many decisions were made only once during this period, which did not allow us to calculate the prior probabilities or proportions, and we had to exclude them from modeling inevitably. For this reason, we have not been able to assess the impact of such policies and decisions on reducing the risk of the disease. Second, in Hamadan, there was not a mechanism to track suspicious and sick people. For this reason, we could not compare our results with other countries or examine the effect of such mechanisms in reducing the risk of COVID-19. Third, one of our problems was the lack of an accurate and up-to-date registration system. Therefore, an expert panel was used in the implementation of the Bayes network model. Although we tried to use experienced individuals in the panel, their opinion may not be completely accurate. It is suggested to use a fuzzy method (quantitative method) instead of the Delphi method (qualitative) to determine conditional probabilities. Fourth, In Hamadan, there was no possibility of full lockdown in this duration. Therefore, it cannot be clearly said that travel restrictions have low effect on reducing the prevalence of this disease. Fifth, one of the obstacles to the progress of the Covid-19 disease is people's knowledge. In this study, we did not have the possibility to measure people's knowledge.

## Conclusion

Choosing the best policy to reduce and control diseases is always challenging and tailored to the circumstances of governments. In the COVID-19 pandemic, these challenges were exacerbated for our government too. According to our results, there is no need to apply many restrictions to control COVID-19. It is possible to control and reduce the risk of COVID-19 in the region with seven policies (i.e., vaccination, limiting gatherings, social distancing, wearing a mask, job closure, travel restriction, and personal hygiene). In other words, this study showed that in contagious pandemics, emphasis on vaccination, avoiding gatherings, physical distance and wearing a mask can greatly reduce the risk of the disease. In areas where strict quarantine is not possible due to economic and technological problems, it is necessary to constantly evaluate different policies and scenarios to control unknown diseases by setting up a comprehensive information registration system.

## Data Availability

The datasets used and/or analyzed during the current study are available from the corresponding author on reasonable request.

## Abbreviations

PMC: Primary health care
CDCC: COVID-19 disease control committee
COVID-19: Coronavirus Disease 2019
SARS-CoV-2: Severe acute respiratory syndrome coronavirus 2

## Footnotes

1 https://coronavirus.jhu.edu/map.html
